# WAVE2 is associated with poor prognosis in pancreatic cancers and promotes cell motility and invasiveness via binding to ACTN4

**DOI:** 10.1002/cam4.1837

**Published:** 2018-10-23

**Authors:** Keisuke Taniuchi, Mutsuo Furihata, Seiji Naganuma, Toshiji Saibara

**Affiliations:** ^1^ Department of Gastroenterology and Hepatology, Kochi Medical School Kochi University Kochi Japan; ^2^ Department of Endoscopic Diagnostics and Therapeutics, Kochi Medical School Kochi University Kochi Japan; ^3^ Department of Pathology, Kochi Medical School Kochi University Kochi Japan

**Keywords:** actin cytoskeleton, ACTN4, cell invasion, p27, pancreatic cancer, WAVE2

## Abstract

WAVE2 is a member of the WASP/WAVE family of actin cytoskeletal regulatory proteins; unfortunately, little is known about its function in pancreatic cancers. In this study, we report the role of WAVE2 in the motility and invasiveness of pancreatic cancer cells. High WAVE2 expression in human pancreatic cancer tissues was correlated with overall survival. WAVE2 accumulated in the cell protrusions of pancreatic cancer cell lines. Downregulation of WAVE2 by small interfering RNA decreased the cell protrusions and inhibited the motility and invasiveness of pancreatic cancer cells. WAVE2 promoted pancreatic cancer cell motility and invasion by forming a complex with the actin cytoskeletal protein alpha‐actinin 4 (ACTN4). Downregulation of ACTN4 by small interfering RNA also inhibited the motility and invasiveness of the cells through a decrease in cell protrusions. Further investigation showed that WAVE2/ACTN4 signaling selectively stimulated p27 phosphorylation and thereby increased the motility and invasiveness of the cells. These results suggest that WAVE2 and ACTN4 stimulate p27 phosphorylation and provide evidence that WAVE2 promotes the motility and invasiveness of pancreatic cancer cells.

## 1 INTRODUCTION

Cell migration and invasion are promoted by actin polymerization at the leading edge of cells and depend on precise coordination of cell protrusion and adhesion.^1^ The WAVE family of proteins, which includes WAVE1, WAVE2, and WAVE3,^2^, ^3^ functions downstream of Ras‐related C3 botulinum toxin substrate 1 (Rac1) for Rac‐induced actin polymerization during lamellipodium formation.^4^ The WAVE2‐Arp2/3 complex causes nucleation of actin assembly,^5^ which leads to lamellipodium formation.^6^ WAVE2 is activated by the Rac1‐IRSp53 signaling pathway^7^ or by binding to Rac1 via a large WAVE2 protein complex that includes Sra1, Nap1, Abi1, and HSPC300.^8^, ^9^ WAVE2 forms a complex with IQ motif containing GTPase activating protein 1 (IQGAP1) and kinesin family member 5B (KIF5B), which is a component of a motor protein complex that mediates the transport of cytoplasmic RNA granules and molecules in migrating breast cancer cells.^10^ When WAVE2 is transported to the cell protrusions with KIF5B, the frequency of cells with lamellipodia increases.^11^

Alpha‐actinin (ACTN) is an actin cross‐linking protein, and four isoforms of ACTN have been identified, including two non‐muscle types, ACTN1 and ACTN4.^12^ ACTN4 is highly localized in the cytoskeleton and is found in the nucleus in response to extracellular stimuli.^13^ ACTN4 promotes cell motility, invasion, and lymph node metastasis in colorectal cancer.^14^ ACTN4 and MDM2 Proto‐Oncogene‐binding protein interact intracellularly, and MDM2 Proto‐Oncogene‐binding protein inhibits ACTN4‐mediated cell migration, the formation of cell protrusions, and actin assembly.^15^, ^16^ ACTN4 influences tumor invasion through its binding to β‐catenin, linking cadherin‐based cell‐cell adhesion and cell locomotion.^17^ In addition, ACTN4 levels significantly correlate with poor prognosis after pancreatic ductal adenocarcinoma (PDAC) resection.^18^ Thus, ACTN4 may contribute to cancer progression through its dynamic actin‐associated stimulation of tumor cell motility, invasion, and metastasis.

The present study aimed to examine WAVE2 expression in tissue samples from PDAC patients and its association with clinicopathological characteristics and survival. We also evaluated the mechanism of WAVE2 in the control of PDAC cell motility and invasion. We showed that WAVE2 induces the formation of cell protrusions through ACTN4 recruitment to filamentous actin in the protrusions and the activation of p27, resulting in an increase in motility and invasiveness of PDAC cells.

## 2 MATERIALS AND METHODS

### 2.1 Antibodies

Anti‐WAVE2 (sc‐373889), anti‐ACTN4 (sc‐390205), and anti‐myc (sc‐789) antibodies were purchased from Santa Cruz (Santa Cruz, CA). JLA20 anti‐actin antibody (MABT219) was purchased from Millipore (Temecula, CA). Anti‐p27 antibody (25614‐1‐AP) was purchased from Proteintech (Chicago, IL). Anti‐phosphorylated p27 antibody (ab85047) was purchased from Abcam (Cambridge, MA).

### 2.2 Primary human PDAC samples

Tumor tissues were obtained from 102 patients who underwent surgical resection of PDAC between 1999 and 2014 at the Department of Surgery of Kochi Medical School Hospital (Nankoku, Japan) or Matsuyama Shimin Hospital (Matsuyama, Japan), as published previously.^19^ The clinical records of all patients were available (Table 1). Tumors were classified according to the Japanese Pancreas Society classification^20^ and the International Union Against Cancer tumor‐node‐metastasis classification (UICC TNM classification).^21^ This study was approved by the ethics review boards of Kochi Medical School and Matsuyama Shimin Hospital prior to patient recruitment. Written informed consent was acquired from each patient prior to initiation.

**Table 1:** Summary of characteristics in 102 patients of pancreatic cancer

Characteristics	Percentage (%)		Characteristics	Percentage (%)	
Age at surgery			Distant metastasis[Link]		
40‐50	3.9	[n = 4]	M0	96.1	[n = 98]
50‐60	16.7	[n = 17]	M1	3.9	[n = 4]
60‐70	31.4	[n = 32]	Histology[Link]		
70‐80	40.2	[n = 41]	PanIN	2.0	[n = 2]
>80	7.8	[n = 8]	Well	30.4	[n = 31]
Gender			Moderate	55.8	[n = 57]
Male	54.9	[n = 56]	Poor	11.8	[n = 12]
Female	45.1	[n = 46]	Venous invasion[Link]		
Stage[Link]			v0	55.4	[n = 57]
0	2.0	[n = 2]	v1	30.7	[n = 31]
IA	3.9	[n = 4]	v2	10.9	[n = 11]
IB	7.8	[n = 8]	v3	3.0	[n = 3]
IIA	31.4	[n = 32]	Lymphatic invasion[Link]		
IIB	49.0	[n = 50]	ly0	42.6	[n = 43]
III	2.0	[n = 2]	ly1	33.6	[n = 34]
IV	3.9	[n = 4]	ly2	19.9	[n = 21]
Primary tumor[Link]			ly3	3.9	[n = 4]
Tis	2.0	[n = 2]	WAVE2 expression		
T1	5.9	[n = 6]	Low	72.5	[n = 74]
T2	14.6	[n = 15]	High	27.5	[n = 28]
T3	75.5	[n = 77]			
T4	2.0	[n = 2]			
Regional lymph nodes[Link]					
N0	45.1	[n = 46]			
N1	54.9	[n = 56]			

PanIN, pancreatic intraepithelial neoplasia.

Classified according to the classification of International Union against Cancer.

Classified according to the classification of pancreatic cancer of Japan Pancreas Society.

### 2.3 Immunohistochemical staining

Immunohistochemistry was carried out using anti‐WAVE2 antibody as published previously.^19^ The immunostaining score was evaluated by two independent observers (SN and MF), who were blinded to the clinical and outcome data. The staining intensity of WAVE2 was scored as: (1) weaker than the intensity of the surface staining of the islets of Langerhans; (2) equal to the intensity of the islets of Langerhans; (3) stronger than the intensity of the islets of Langerhans. The proportion of tumor cells were graded from 1 to 3:1 (<50%), 2 (50%‐80%), and 3 (>80%). The total immunohistochemical score was calculated by summing the percentage score and the intensity score. WAVE2 expression was classified into two groups based on the total score (low group, 2‐3; high group, 4‐6) with reference to previous reports.^19^, ^22^

### 2.4 Cell culture

The human PDAC cell line S2‐013, a subline of SUIT‐2, was obtained from Dr T. Iwamura (Miyazaki Medical College, Miyazaki, Japan).^23^ The human PDAC cell line PANC‐1 was purchased from the American Type Culture Collection (Manassas, VA). HPNE immortalized normal pancreatic epithelial cells were a kind gift from Dr Michel Ouellette (University of Nebraska Medical Center, Omaha, NE).^24^ These cell lines were maintained in Dulbecco's modified Eagle's medium (Gibco‐BRL, Carlsbad, CA) containing 10% fetal calf serum.

### 2.5 Confocal immunofluorescence microscopy

Immunocytochemistry was carried out as published previously.^25^

### 2.6 Small interfering RNA (siRNA) treatment

A single mixture with four different siRNA oligonucleotides targeting *WAVE2*, one with four different siRNA oligonucleotides targeting *ACTN4,* and one with four different siRNA oligonucleotides targeting *p27* were purchased from Qiagen (FlexiTube GeneSolution GS10163, GS1027, and GS81, respectively; Valencia, CA), and a single mixture with four different scrambled negative control siRNA oligonucleotides was obtained from Santa Cruz (37007). S2‐013 and PANC‐1 cells were transfected with each siRNA mixture in siRNA transfection reagent (Qiagen) following the manufacturer's instructions. After incubation for 48 hours, total cell lysates were extracted, and immunoblotting was carried out to evaluate the effects of siRNA treatment.

### 2.7 WAVE2 rescue construct

The entire coding sequence of the *WAVE2* cDNA was cloned into pCMV6‐Entry vector (Origene Technologies, Rockville, MD) bearing a C‐terminal myc‐DDK‐tag by reverse transcription polymerase chain reaction of total RNA extracted from S2‐013 cells. Transient transfection of the resulting *WAVE2* rescue construct was carried out with X‐tremeGENE HP DNA Transfection Reagent (Roche, Penzberg, Germany). The transfected cells were typically assayed 2 days after transfection.

### 2.8 Transwell motility assay

The Transwell motility assay was carried out as published previously.^25^ The assay was performed three independent times.

### 2.9 Matrigel invasion assay

The Matrigel invasion assay was carried out as published previously.^25^ The assay was performed three independent times.

### 2.10 In vitro growth rate as determined with the 3‐(4,5‐dimethylthiazol‐2‐yl)‐2,5‐diphenyltetrazolium bromide (MTT) assay

S2‐013 and PANC‐1 cells transiently transfected with scrambled negative control siRNA, *WAVE2* siRNA, *ACTN4* siRNA, or *p27* siRNA were each seeded at a concentration of 5 × 10^4^ cells per well in 12‐well plates. The viability of the cells was evaluated with the MTT assay according to the manufacturer's instructions. Briefly, 1/10 volume cell counting kit‐8 solution (Dojindo, Kumamoto, Japan) was added to each well, and the plates were incubated at 37°C for 3 hours. Absorbance was then measured at 490 nm and at 630 nm as a reference, with a Microplate Reader 550 (Bio‐Rad, Hercules, CA).

### 2.11 Immunoprecipitation and mass spectrometric analysis of WAVE2

Combined immunoprecipitation and mass spectrometric analysis using a nano‐LC‐MS/MS system (Genomine, Inc, Pohang, Korea) were performed as published previously.^26^

### 2.12 Immunoprecipitation

S2‐013 cells were incubated on fibronectin for 5 hours and lysed in lysis buffer (50 mmol/L Tris [pH 7.4], 150 mmol/L NaCl, 1 mmol/L MgCl_2_, 0.5% NP‐40, and protease inhibitor cocktail tablets [Roche], and phosphatase inhibitor cocktail [Nacalai, Kyoto, Japan]). The resulting lysates were immunoprecipitated with anti‐WAVE2 antibody, anti‐ACTN4 antibody, or mouse IgG isotype control antibody, and Dynabeads Protein G (Dynal, Oslo, Norway). After the beads were subsequently washed with wash buffer (50 mmol/L Tris [pH 7.4], 150 mmol/L NaCl, 1 mmol/L MgCl_2_, and 0.5% NP‐40), immune complexes were analyzed on Western blots to examine the interaction between endogenous WAVE2 and ACTN4.

### 2.13 Cell fractionation

Nuclear and cytoplasmic fractionation was performed using a LysoPure Nuclear and Cytoplasmic Extractor Kit (Wako, Osaka, Japan) as previously described.^27^, ^28^

### 2.14 Phospho‐kinase array assay

The Proteome Profiler Human Phospho‐Kinase Array Kit ARY003 was purchased from R&D Systems (Minneapolis, MN) and used according to the manufacturer's protocol, as published previously.^29^ Blots were quantified by densitometric analysis using Kodak EDAS290 image analysis software (Kodak, Rochester, NY).

### 2.15 Statistical analysis

For immunohistochemical analysis, we performed statistical analysis using R (version 3.3.3; The R Foundation, Wien, Austria) as published previously.^19^ Fisher's exact test was used to assess the correlation between WAVE2 expression levels and clinicopathological parameters. The Kaplan‐Meier method and log‐rank test (Mantel‐Cox) were carried out to calculate cumulative survival rates. Survival rates are expressed as the median value and interquartile range. Univariate Cox regression analysis was performed to determine the prognostic significance of individual clinicopathological factors. Cox proportional hazards models were used for multivariate analysis of independent factors for overall survival. For the in vitro experiments, statistical significance was evaluated with Student's *t* tests. *P* values <0.05 were considered significant and indicated with asterisks in the figures.

## 3 RESULTS

### 3.1 WAVE2 expression in PDAC tissue samples

**Figure 1:**
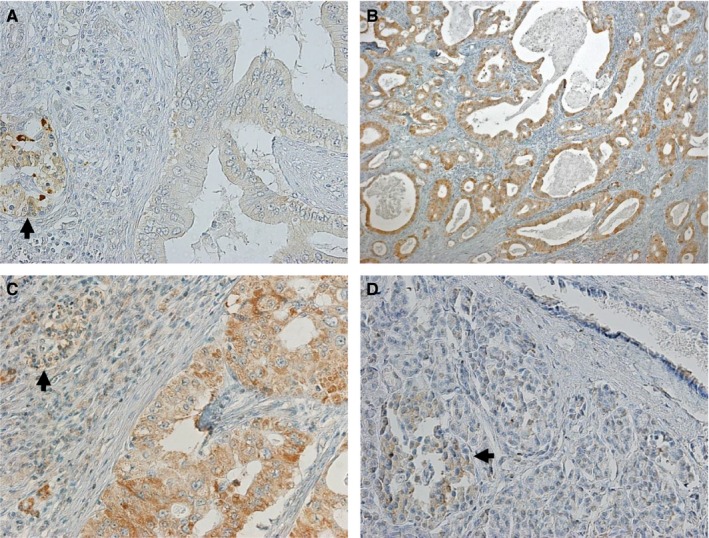
Immunohistochemistry with anti‐WAVE2 antibody. A, Representative immunohistochemical staining of PDAC tissues using anti‐WAVE2 antibody showing low expression of WAVE2. Arrow, the islet of Langerhans. Magnification: ×200. B and C, Representative immunohistochemical staining of PDAC tissues using anti‐WAVE2 antibody showing high expression of WAVE2. Arrow, the islet of Langerhans. Magnification: ×40 (B) and ×200 (C). D, Expression of WAVE2 in the normal pancreas. Arrow, the islet of Langerhans. Magnification: ×200

The WAVE2 expression levels were examined in surgical specimens from 102 patients with PDAC by immunohistochemical staining (Table 1). Immunostaining scores were used to classify patients into the low‐expressing WAVE2 group (72.5%; n = 74; total immunohistochemical score = 2 or 3; Figure 1A) and the high‐expressing WAVE2 group (27.5%; n = 28; total immunohistochemical score = 4, 5, or 6; Figure 1B,C). WAVE2 was not obviously present in normal pancreatic ducts (Figure 1D), brain, lung, liver, or kidney (data not shown).

### 3.2 Associations of WAVE2 overexpression with clinicopathological features and prognosis

The relationship between the WAVE2 expression level in PDAC tissue samples and clinicopathological variables is shown in Table 2. The overall expression score was not significantly correlated with clinicopathological variables.

**Table 2:** Correlation between WAVE2 expression and clinicopathological parameters

	WAVE2 expression				*P*
	Low		High		
	Percentage (%)				
Stage[Link]					0.621
0	2.7	[n = 2]	0	[n = 0]	
IA	2.7	[n = 2]	7.1	[n = 2]	
IB	9.5	[n = 7]	3.6	[n = 1]	
IIA	29.7	[n = 22]	35.7	[n = 10]	
IIB	50.0	[n = 37]	46.4	[n = 13]	
III	2.7	[n = 2]	0	[n = 0]	
IV	2.7	[n = 2]	7.1	[n = 2]	
Primary tumor[Link]					0.605
Tis	2.7	[n = 2]	0	[n = 0]	
T1	4.1	[n = 3]	10.7	[n = 3]	
T2	16.2	[n = 12]	10.7	[n = 3]	
T3	74.3	[n = 55]	78.6	[n = 22]	
T4	2.7	[n = 2]	0	[n = 0]	
Regional lymph nodes[Link]					0.373
N0	41.9	[n = 31]	53.6	[n = 15]	
N1	58.1	[n = 43]	46.4	[n = 13]	
Distant metastasis[Link]					0.302
M0	97.3	[n = 72]	92.9	[n = 26]	
M1	2.7	[n = 2]	7.1	[n = 2]	
Histology[Link]					0.598
PanIN	2.7	[n = 2]	0	[n = 0]	
Well	31.1	[n = 23]	28.5	[n = 8]	
Moderate	56.7	[n = 42]	53.6	[n = 15]	
Poor	9.5	[n = 7]	17.9	[n = 5]	
Venous invasion[Link]					0.2
v0 + v1	89.2	[n = 66]	78.6	[n = 22]	
v2 + v3	10.8	[n = 8]	21.4	[n = 6]	
Lymphatic invasion[Link]					0.798
ly0 + ly1	74.3	[n = 55]	78.6	[n = 22]	
ly2 + ly3	25.7	[n = 19]	21.4	[n = 6]	

PanIN, pancreatic intraepithelial neoplasia.

Classified according to the classification of International Union against Cancer

**Figure 2:**
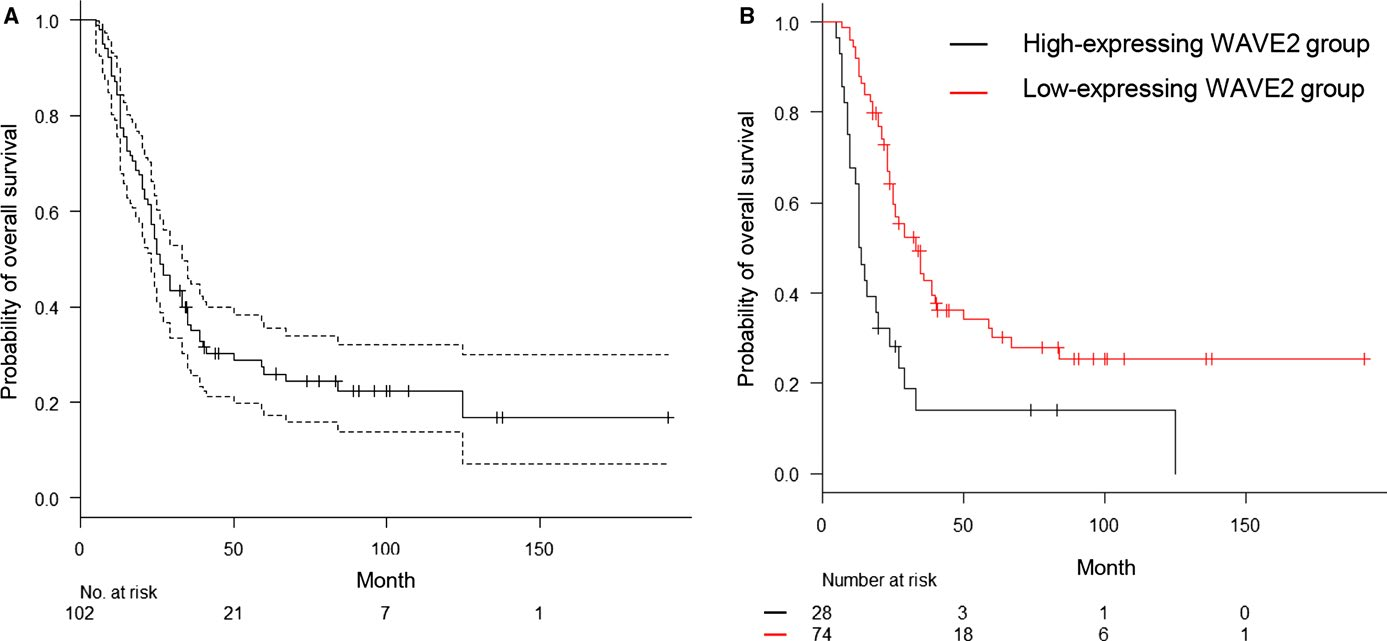
Correlation between high expression of WAVE2 and poor outcome in PDAC patients. (A) Kaplan‐Meier analysis of PDAC‐specific survival and (B) overall survival according to WAVE2 expression. The dashed lines represent the lower and upper limits of the 95% confidence interval

Classified according to the classification of pancreatic cancer of Japan Pancreas Society;

The overall survival time for postoperative PDAC patients with WAVE2 overexpression was significantly shorter than that of PDAC patients with low WAVE2 expression (Figure 2A,B, Kaplan‐Meier curves, *P* < 0.001). We examined the prognostic value of WAVE2 expression in subgroups stratified by UICC stage, age, gender, tumor size, differentiation grade, lymphatic invasion, venous invasion, and intrapancreatic nerve invasion. Univariate Cox regression analysis revealed that stage III and IV, high WAVE2 expression, tumor size, and venous invasion were independent prognostic factors (Table 3). Furthermore, multivariate analysis revealed that stage III and IV and high WAVE2 expression were independent factors of worse overall survival (Table 3).

**Table 3:** Univariate and multivariate analysis of prognostic factors for overall survival

	Overall survival			
	Univariate		Multivariate	
	HR (95% CI)	*P*	HR (95% CI)	*P*
Stage[Link]				
0 + IA + IB	1.0 (reference)		1.0 (reference)	
IIA	1.159 (0.714‐1.881)	0.549	4.475 (1.541‐12.99)	0.006
IIB	1.356 (0.854‐2.151)	0.196	5.181 (1.834‐14.64)	0.002
III + IV	3.035 (1.301‐7.081)	0.010	13.96 (3.835‐50.81)	6.361e‐05
Age	1.021 (0.995‐1.048)	0.110	1.027 (0.999‐1.054)	0.050
Gender	1.107 (0.696‐1.761)	0.666	1.280 (0.795‐2.060)	0.310
WAVE2 expression	0.398 (0.242‐0.653)	2.662e‐04	0.376 (0.226‐0.627)	1.746e‐04
Diameter of primary tumor	1.338 (1.176‐1.524)	1.045e‐05		
Histology[Link]	1.383 (0.846‐2.261)	0.196		
Lymphatic invasion[Link] (ly0 + ly1 or ly2 + ly3)	1.269 (0.751‐2.145)	0.373		
Venous invasion[Link] (v0 + v1 or v2 + v3)	1.928 (1.034‐3.593)	0.038		
Intrapancreatic nerve invasion[Link] (n0 + n1 or n2 + n3)	1.500 (0.947‐2.377)	0.083		

Classified according to the classification of International Union against Cancer;

Classified according to the classification of pancreatic cancer of Japan Pancreas Society

### 3.3 Subcellular distribution of WAVE2 in PDAC cells grown on fibronectin

**Figure 3:**
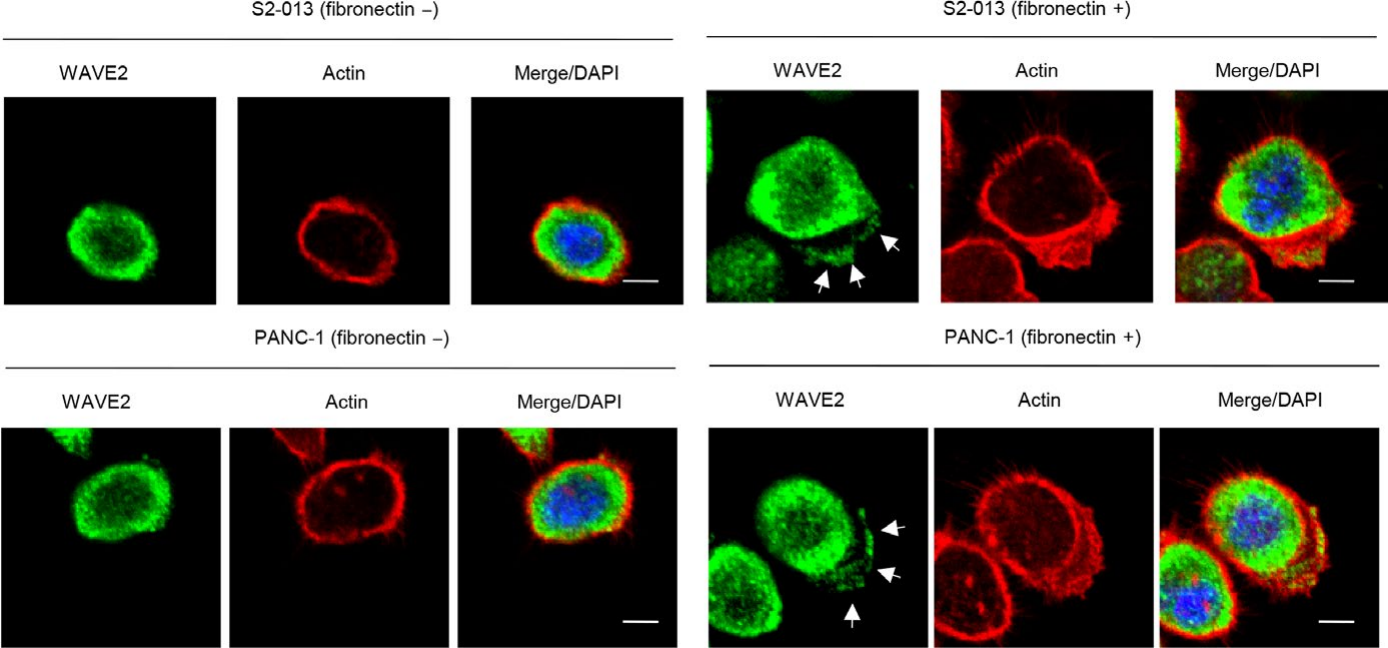
Subcellular localization of WAVE2 in PDAC cells grown on fibronectin. Confocal immunofluorescence microscopic images. S2‐013 cells were cultured on fibronectin and then labeled with anti‐WAVE2 antibody (green). Actin filaments were labeled with phalloidin (red). Arrows, WAVE2 localized in cell protrusions. Blue, DAPI staining. Scale bar, 10 μm

Immunocytochemistry was used to investigate the subcellular localization of WAVE2 in the moderately differentiated S2‐013 PDAC cells and the poorly differentiated PANC‐1 PDAC cells. Fibronectin induces the formation of cell protrusions at the leading edge of PDAC cells.^25^ S2‐013 and PANC‐1 cells formed fewer cell protrusions when cultured without fibronectin than when cultured on fibronectin.^25^ In S2‐013 and PANC‐1 cells grown on fibronectin, WAVE2 was present in the cytoplasm of the cell bodies as well as in the cell protrusions, each of which contained many peripheral actin structures (Figure 3).

### 3.4 Roles of WAVE2 in the motility and invasiveness of PDAC cells

**Figure 4:**
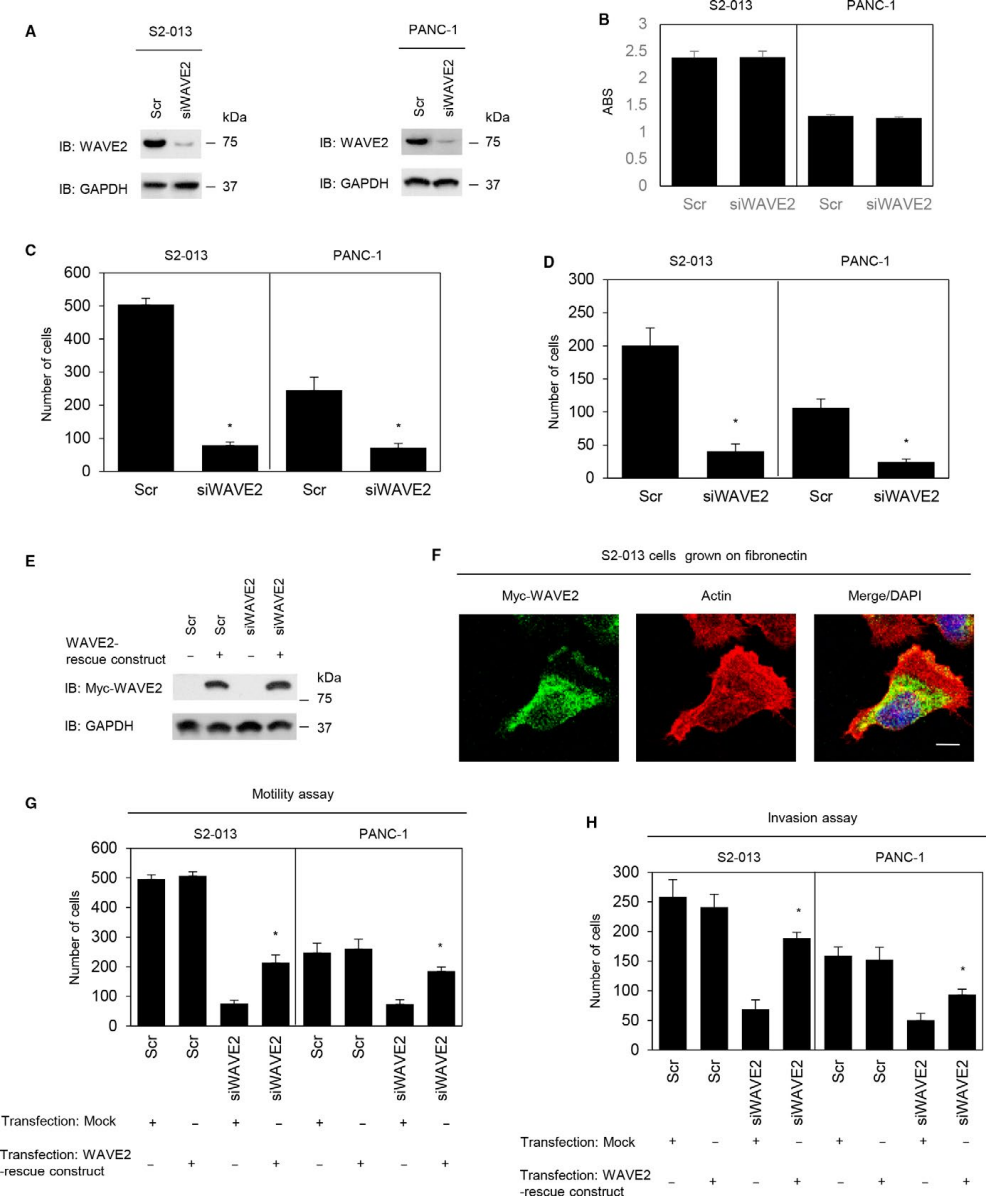
Roles of WAVE2 in the motility and invasiveness of PDAC cells. A, siRNA oligonucleotides targeting *WAVE2* (siWAVE2) or negative control scrambled siRNAs (Scr) were transiently transfected into S2‐013 and PANC‐1 cells. Western blotting was performed using anti‐WAVE2 antibody. B, MTT assays of S2‐013 and PANC‐1 cells transiently transfected with scrambled control siRNA or *WAVE2* siRNA were performed to evaluate cell viability. Data are representative of three independent experiments and are the means ±SD. ABS on *Y*‐axis means absorbance at 490 nm and at 630 nm as reference measured with a microplate reader. C and D, Scr or siWAVE2 was transiently transfected into S2‐013 and PANC‐1 cells. Motility (C) and two‐chamber invasion (D) assays were performed. Migrating cells in four fields per group were scored. Data were derived from three independent experiments. *Columns*, mean; *bars*, standard deviation (SD). *P < 0.005 compared to the Scr‐transfected control (Student's *t* test). E, A mock control vector or myc‐tagged WAVE2 rescue construct was transiently transfected into Scr control siRNA‐transfected and *WAVE2* siRNA‐transfected S2‐013 cells. Myc‐tagged WAVE2 was detected by Western blotting with anti‐myc antibody. F, Confocal immunofluorescence microscopic images. The myc‐tagged WAVE2 rescue construct was transfected into S2‐013 cells; 48 h later, the cells were incubated on fibronectin. The cells were stained with anti‐myc antibody (green). Actin filaments were labeled with phalloidin (red). Blue, DAPI staining. Scale bar, 10 µm. G and H, A mock control vector or myc‐tagged WAVE2 rescue construct was transiently transfected into Scr control siRNA‐transfected and *WAVE2* siRNA‐transfected S2‐013 and PANC‐1 cells; 48 h later, motility (F) and two‐chamber invasion (G) assays were performed. Migrating cells in four fields per group were counted. Data were derived from three independent experiments. *Columns*, mean; *bars*, SD. *P < 0.008 compared to the corresponding *WAVE2* siRNA‐transfected cells that were transfected with the mock vector (Student's *t* test).

WAVE2 was transiently suppressed by transfection with *WAVE2* siRNA in S2‐013 and PANC‐1 cells (Figure 4A). An in vitro MTT assay showed that suppression of WAVE2 did not affect cell growth in S2‐013 and PANC‐1 cells (Figure 4B). Suppression of WAVE2 significantly inhibited cell motility (Figure 4C) and invasion (Figure 4D) in S2‐013 and PANC‐1 cells. The expression of myc‐tagged rescue WAVE protein was confirmed in scrambled control siRNA‐transfected and *WAVE2* siRNA‐transfected S2‐013 cells (Figure 4E). Similar to endogenous WAVE2, the exogenous rescue WAVE protein was localized in the cytoplasm of the cell bodies and in the cell protrusions of S2‐013 cells (Figure 4F). The rescue WAVE protein restored cell motility and invasiveness that was inhibited after WAVE2 silencing in S2‐013 and PANC‐1 cells (Figure 4G,H). Thus, our data indicate that WAVE2 promoted the motility and invasiveness of PDAC cells.

### 3.5 Association of WAVE2 with ACTN4

**Figure 5:**
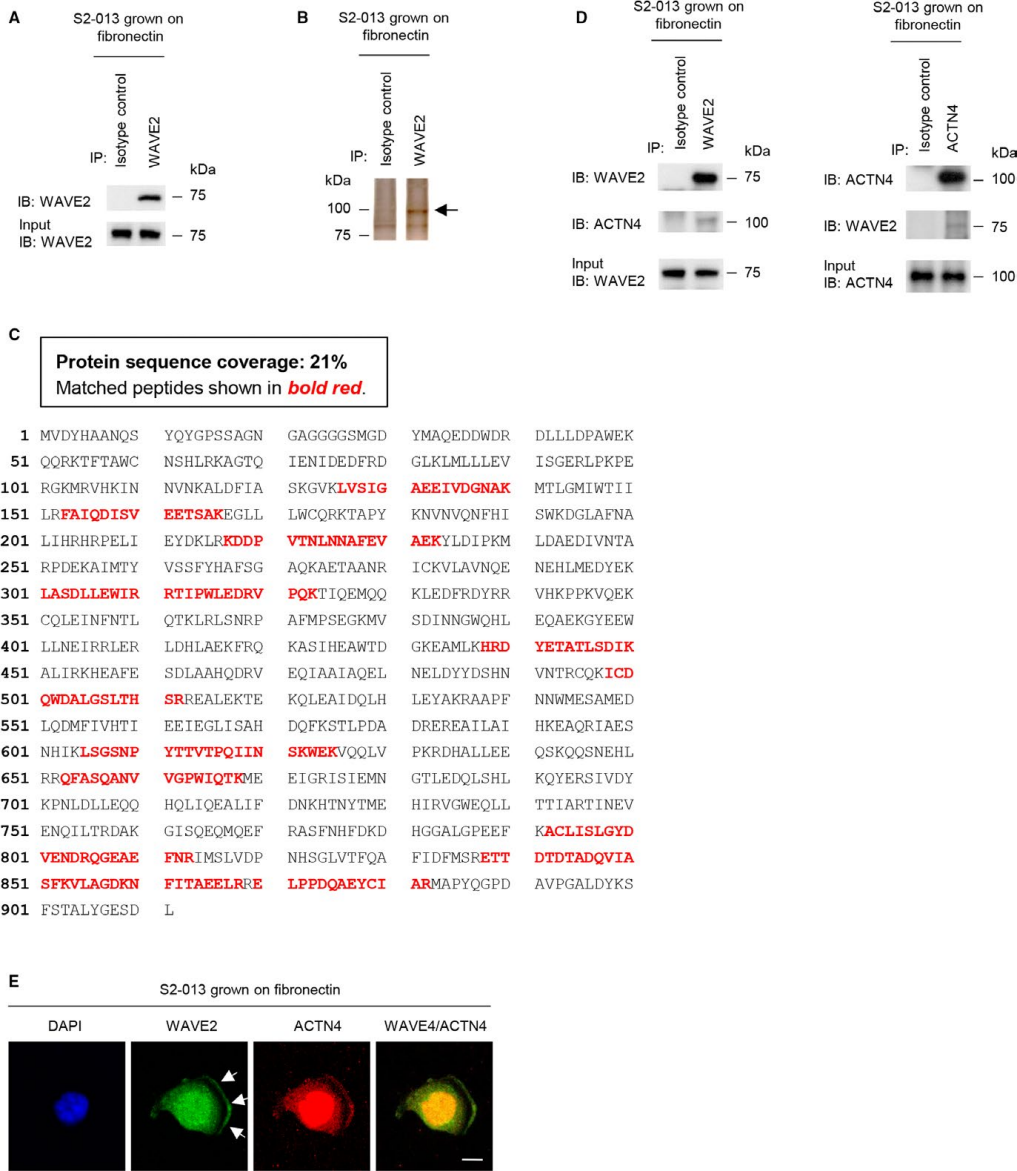
Association of WAVE2 with ACTN4. A, Immunoprecipitation of WAVE2 from S2‐013 cells cultured on fibronectin. Rabbit IgG isotype control antibody was used as the control. Proteins within immunoprecipitates were examined on Western blots probed with anti‐WAVE2 antibody. B, Proteins in immunoprecipitates were examined with silver staining. Rabbit IgG isotype control antibody was used as the control. A 100‐kDa band is indicated by the arrow. C, The percent coverage for ACTN4 is represented by the identified peptides in the total protein sequence (accession number NP_004915). D, Immunoprecipitation of WAVE2 or ACTN4 from S2‐013 cells cultured on fibronectin. Proteins within immunoprecipitates were examined on Western blots probed with antibodies against WAVE2 and ACTN4. Rabbit IgG isotype control antibody for WAVE2 and mouse IgG isotype control antibody for ACTN4 was used as controls. E, Confocal immunofluorescence microscopic images. S2‐013 cells were cultured on fibronectin and then labeled with anti‐WAVE2 (green) and anti‐ACTN4 (red) antibodies. Arrows, WAVE2 co‐localized with ACTN4 in cell protrusions. Blue, DAPI staining. Scale bars, 10 µm

To explore WAVE2‐specific mechanisms in cell motility and invasiveness, immunoprecipitation experiments were performed to detect potential WAVE2‐binding proteins. WAVE2 immunoprecipitated with the anti‐WAVE2 antibody (Figure 5A). Following SDS‐PAGE and silver staining, a specific 100‐kDa protein was detected in the anti‐WAVE2 immunoprecipitates (Figure 5B). The protein band was excised, subjected to LC‐MS/MS analysis, and identified as ACTN4. The peptide sequence coverage was 21% (Figure 5C). The interaction between WAVE2 and ACTN4 was confirmed using co‐immunoprecipitation experiments in S2‐013 cells grown on fibronectin (Figure 5D). Immunocytochemistry showed that WAVE2 and ACTN4 were co‐localized in the cytoplasm and nucleus, and a portion of WAVE2 and ACTN4 accumulated in the cell protrusions of S2‐013 cells grown on fibronectin (Figure 5E).

### 3.6 Roles of WAVE2 in the co‐localization of ACTN4 with actin polymerization

**Figure 6:**
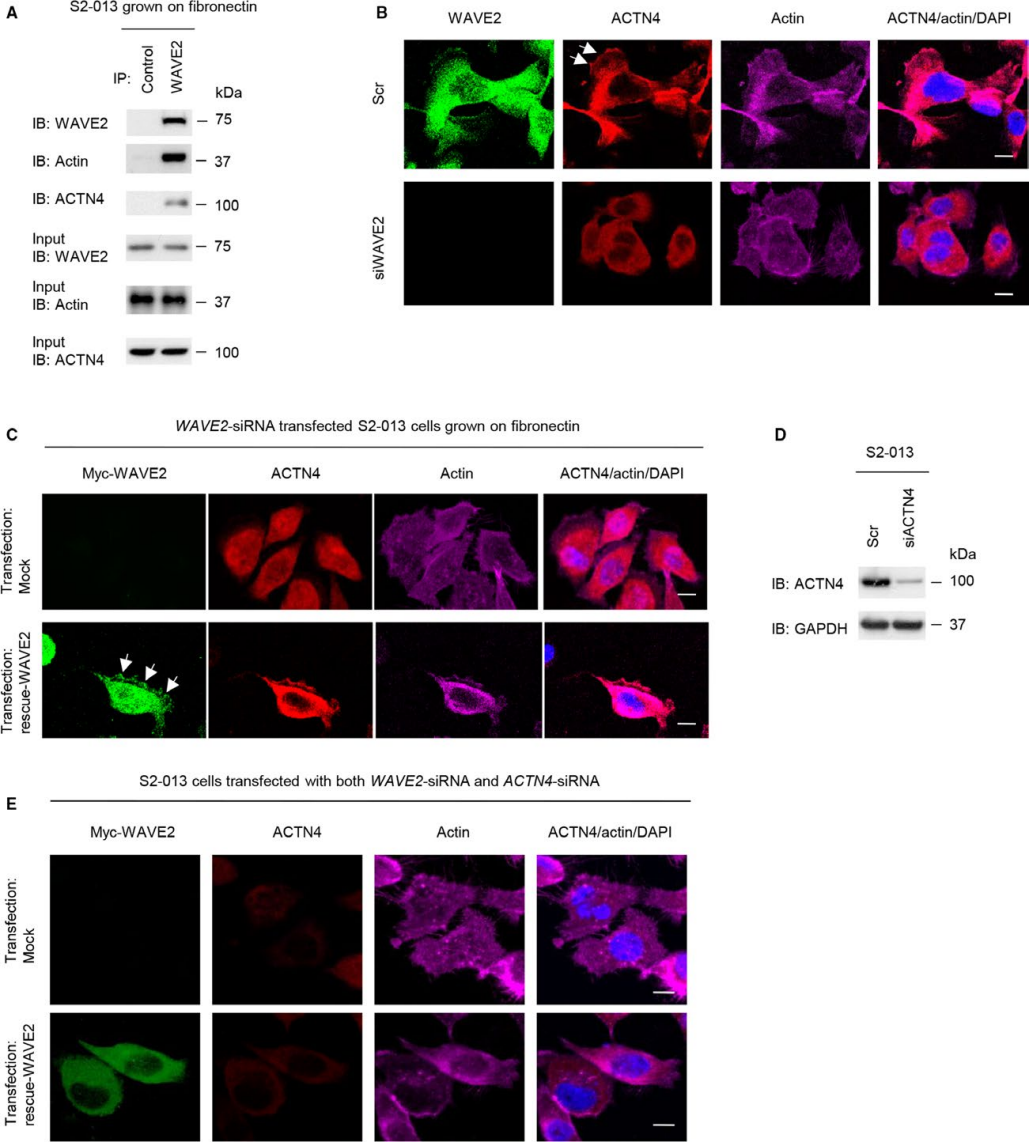
Roles of WAVE2 in the translocation of ACTN4 to actin filaments in cell protrusions. A, Immunoprecipitation of WAVE2 from S2‐013 cells cultured on fibronectin. Proteins within immunoprecipitates were examined on Western blots probed with antibodies against WAVE2, ACTN4, and actin. Rabbit IgG isotype control antibody was used as the control. B, Confocal immunofluorescence microscopic images. Oligonucleotides (siRNAs targeting *WAVE2* (siWAVE2) or scrambled siRNAs (Scr) as the negative control) were transiently transfected into S2‐013 cells. Transfected cells were incubated on fibronectin and were subsequently stained with anti‐WAVE2 antibody (green), anti‐ACTN4 antibody (red), and phalloidin (violet). Arrows, ACTN4 bound to peripheral actin structures in cell protrusions. Blue, DAPI staining. Scale bars, 10 µm. C, Confocal immunofluorescence microscopic images. A myc‐tagged WAVE2 rescue construct was transfected into S2‐013 cells that had been transfected with *WAVE2* siRNA; 48 h later, cells were incubated on fibronectin. Cells were stained with anti‐myc antibody (green), anti‐ACTN4 antibody (red), and phalloidin (violet). Arrows, exogenous WAVE2 localized in cell protrusions. Blue, DAPI staining. Scale bar, 10 µm. D, Oligonucleotides (siRNAs targeting *ACTN4* (siACTN4) or Scr) were transiently transfected into S2‐013 cells. Western blotting was performed using anti‐ACTN4 antibody. E, Confocal immunofluorescence microscopic images. A myc‐tagged WAVE2 rescue construct was transfected into S2‐013 cells that had been transfected with both *WAVE2* siRNA and *ACTN4* siRNA; 48 h later, cells were incubated on fibronectin. Cells were stained with anti‐myc antibody (green), anti‐ACTN4 antibody (red), and phalloidin (violet). Blue, DAPI staining. Scale bars, 10 µm

Immunoprecipitation experiments showed the interaction between WAVE2 and filamentous actin in S2‐013 cells grown on fibronectin (Figure 6A), indicating that actin was enriched in the WAVE2‐immunoprecipitated materials containing ACTN4. Immunocytochemistry showed that the level of ACTN4 bound to the actin cytoskeleton was decreased in *WAVE2* siRNA‐transfected S2‐013 cells grown on fibronectin in comparison with the scrambled control (Figure 6B). *WAVE2* siRNA‐transfected S2‐013 cells expressing the rescue WAVE protein showed restored expression of ACTN4 bound to the actin cytoskeleton that was decreased after WAVE2 silencing (Figure 6C).

ACTN4 was transiently suppressed by transfection with *ACTN4* siRNA in S2‐013 cells (Figure 6D). Transfection of a WAVE2 rescue construct into *ACTN4* siRNA‐transfected S2‐013 and PANC‐1 cells in which WAVE2 had been suppressed did not increase the actin‐driven membrane protrusions compared to S2‐013 cells without the rescue WAVE protein (Figure 6E).

### 3.7 Roles of WAVE2 and ACTN4 in forming cell protrusions

**Figure 7:**
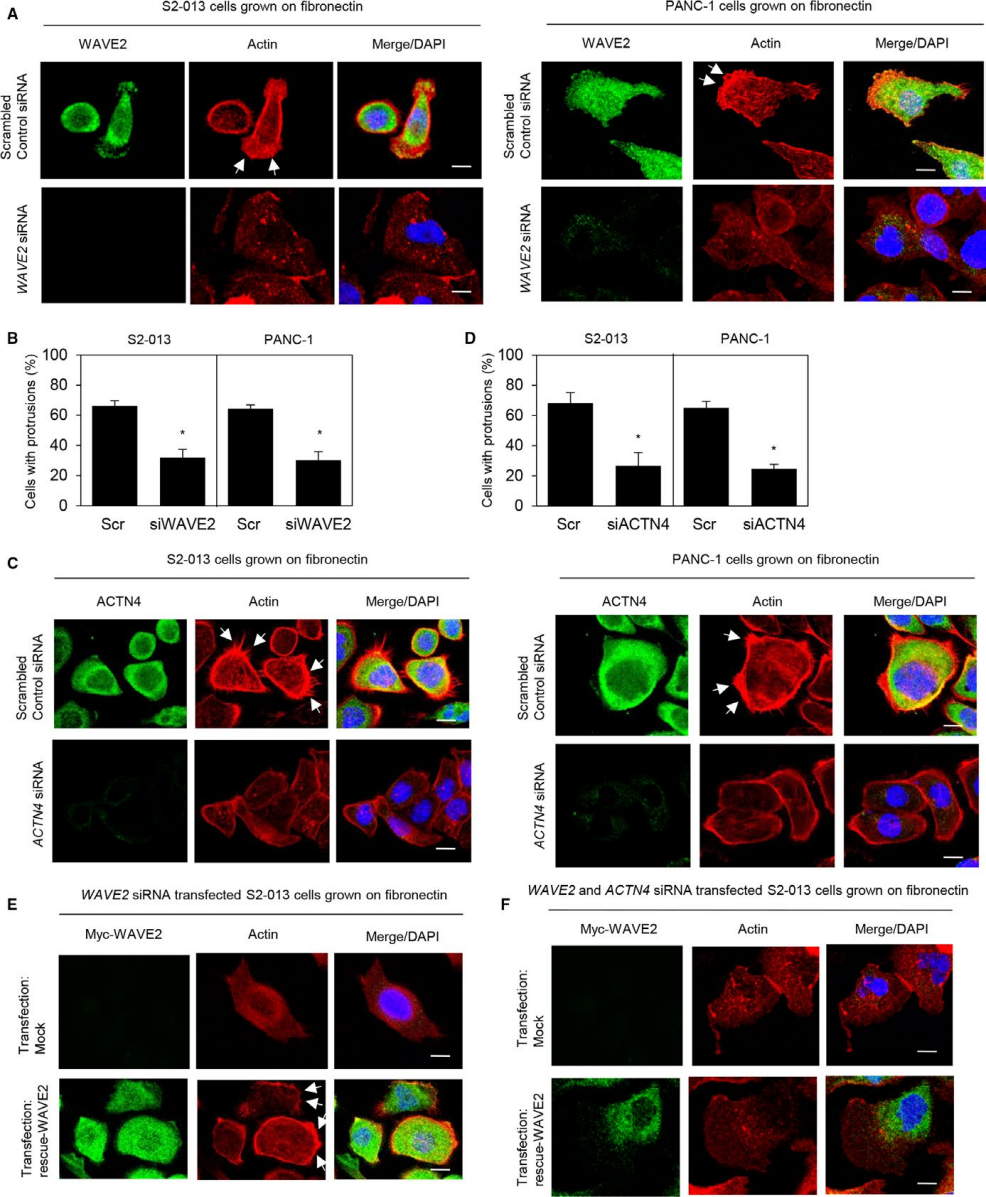
Roles of WAVE2 and ACTN4 in forming cell protrusions. A, Confocal immunofluorescence microscopic images showing phalloidin‐labeled peripheral actin structures (red) and DAPI‐labeled nuclei (blue) in scrambled control siRNA‐transfected S2‐013 and PANC‐1 cells, and *WAVE2* siRNA‐transfected S2‐013 and PANC‐1 cells grown on fibronectin. Arrows, peripheral actin structures in cell protrusions. Scale bars, 10 µm. B, Quantification of data shown in A; the values represent the number of cells with protrusions in which the levels of peripheral actin structures were increased. All cells in four fields per group were scored. Data were derived from three independent experiments. *Columns*, mean; *bars*, SD. *P < 0.001 compared to the scrambled control siRNA‐transfected S2‐013 and PANC‐1 cells (Student's *t* test). C, Confocal immunofluorescence microscopic images showing phalloidin‐labeled peripheral actin structures (red) and DAPI‐labeled nuclei (blue) in scrambled control siRNA‐transfected S2‐013 and PANC‐1 cells, and *ACTN4* siRNA‐transfected S2‐013 and PANC‐1 cells grown on fibronectin. Arrows, peripheral actin structures in cell protrusions. The lower and right panels in the confocal Z stack show a vertical cross section (yellow lines) through the cells. Bars, 10 µm. D, Quantification of the data shown in C; the values represent the number of cells with protrusions in which the levels of peripheral actin structures were increased (right panel). All cells in four fields per group were scored. Data were derived from three independent experiments. *Columns*, mean; *bars*, SD. *P < 0.001 compared to the scrambled control siRNA‐transfected S2‐013 and PANC‐1 cells (Student's *t* test). E, Confocal immunofluorescence microscopic images. The mock control vector or myc‐tagged WAVE2 rescue construct was transiently transfected into scrambled control siRNA‐transfected and *WAVE2* siRNA‐transfected S2‐013 and PANC‐1 cells; 48 h later, the cells were incubated on fibronectin. Cells were stained with anti‐myc antibody (green). Actin filaments were labeled with phalloidin (red). Arrows, cell protrusions recovered by exogenous WAVE2 in *WAVE2* siRNA‐transfected cells. Blue, DAPI staining. Scale bars, 10 µm. F, Confocal immunofluorescence microscopic images. The myc‐tagged WAVE2 rescue construct was transfected into S2‐013 and PANC‐1 cells that had been transfected with *WAVE2* siRNA and *ACTN4* siRNA; 48 h later, the cells were incubated on fibronectin. Cells were stained with anti‐myc antibody (green) and phalloidin (red). Blue, DAPI staining. Scale bars, 10 µm.

We analyzed actin polymerization in the cell protrusions of S2‐013 and PANC‐1 cells transfected with *WAVE2* siRNA. Compared with the scrambled control, suppression of WAVE2 decreased actin polymerization in the cell protrusions and significantly inhibited the formation of protrusions (Figure 7A,B). Suppression of ACTN4 also decreased the level of actin polymerization in the cell protrusions in comparison with the scrambled control (Figure 7C,D). The rescue WAVE protein restored the cell protrusions with peripheral actin assembly that was inhibited after WAVE2 silencing in S2‐013 and PANC‐1 cells (Figure 7E). Transfection of a WAVE2 rescue construct into *ACTN4* siRNA‐transfected S2‐013 and PANC‐1 cells in which WAVE2 had been suppressed did not restore the actin‐driven membrane protrusions compared to cells without the rescue WAVE protein (Figure 7F). These results indicated that WAVE2 and ACTN4 cooperatively induce the actin‐driven membrane protrusions in PDAC cells.

### 3.8 Roles of WAVE2 and ACTN4 in the motility and invasiveness of PDAC cells

**Figure 8:**
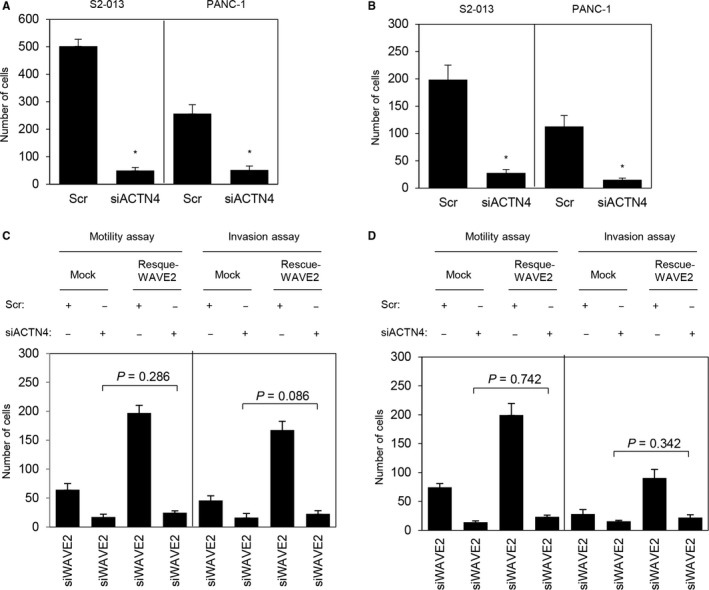
Roles of WAVE2 and ACTN4 in the motility and invasiveness of PDAC cells. A and B, siACTN4 oligonucleotides or Scr oligonucleotides were transiently transfected into S2‐013 and PANC‐1 cells. Motility (A) and two‐chamber invasion (B) assays were performed. Migrating cells in four fields per group were scored. Data were derived from three independent experiments. *Columns*, mean; *bars*, SD. *P < 0.004 compared to the Scr‐transfected cells (Student's *t* test). C and D, The myc‐tagged WAVE2 rescue construct was transfected into S2‐013 (C) and PANC‐1 (D) cells that had been transfected with *WAVE2* siRNA and *ACTN4* siRNA; 48 h later, motility and two‐chamber invasion assays were performed. Migrating cells in four fields per group were counted. Data were derived from three independent experiments. *Columns*, mean; *bars*, SD.

Silencing of ACTN4 significantly reduced cell motility (Figure 8A) and invasion (Figure 8B) of S2‐013 and PANC‐1 cells. Transfection of a WAVE2 rescue construct into *ACTN4* siRNA‐transfected S2‐013 and PANC‐1 cells in which WAVE2 had been suppressed did not rescue cell motility and invasion after silencing of WAVE2 and ACTN4 (Figure 8C,D). These results indicated that WAVE2 and ACTN4 cooperatively promote the motility and invasiveness of PDAC cells.

### 3.9 Association of WAVE2 and ACTN4 with cell signaling pathways

**Figure 9:**
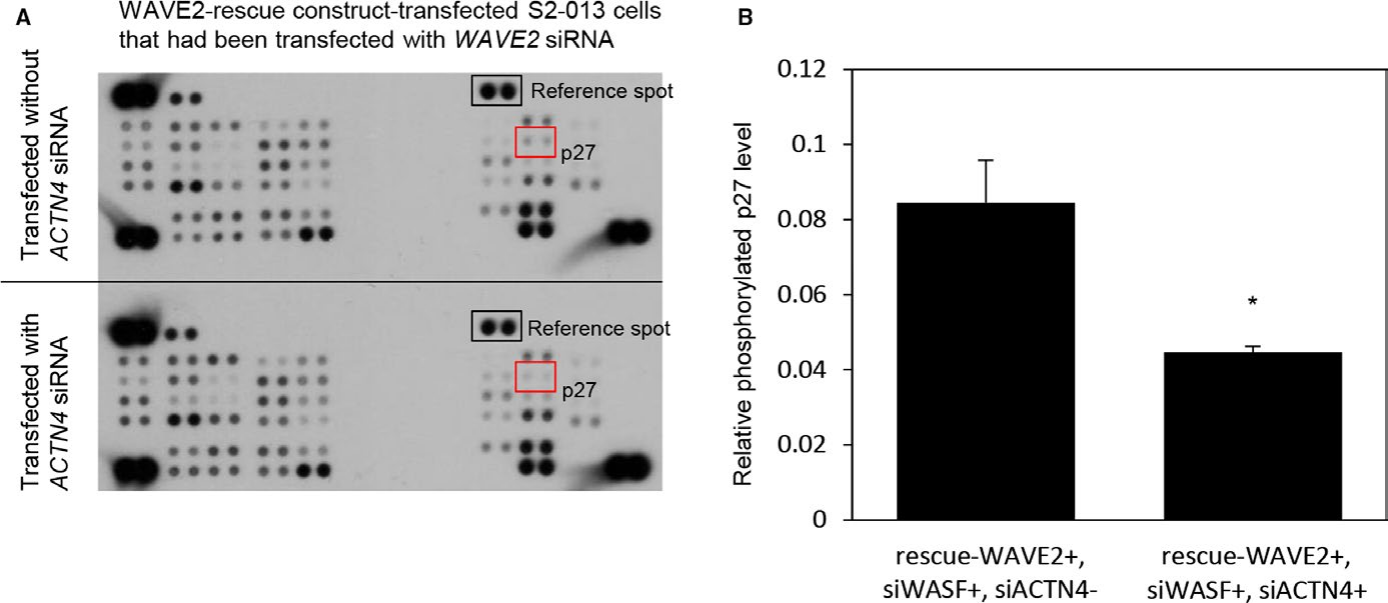
Signaling pathway molecules associated with WAVE2 and ACTN4. A, Human phosphoprotein arrays showing the differential phosphorylation of proteins between WAVE2 rescue construct‐transfected S2‐013 cells that had been transfected with both *WAVE2* siRNA and *ACTN4* siRNA, and WAVE2 rescue construct‐transfected S2‐013 cells that had been transfected with *WAVE2* siRNA alone. Data are representative of three independent experiments. B, Densitometric analysis of the results of A. The level of phosphorylated p27 in WAVE2 rescue construct‐transfected S2‐013 cells that had been transfected with *WAVE2* siRNA alone was compared to that in WAVE2 rescue construct‐transfected S2‐013 cells that had been transfected with both *WAVE2* siRNA and *ACTN4* siRNA. Data are derived from three independent experiments. *Columns*, mean; *bars*, SD. *P < 0.01 compared with WAVE2 rescue construct‐transfected S2‐013 cells that had been transfected with both *WAVE2* siRNA and *ACTN4* siRNA (Student's *t* test).

To determine whether WAVE2 and ACTN4 could regulate the activity of phosphoproteins, we performed a phosphoprotein array analysis and compared WAVE2 rescue construct‐transfected S2‐013 cells with suppression of both WAVE2 and ACTN4, with WAVE2 rescue construct‐transfected S2‐013 cells with suppression of WAVE2 alone (Figure 9A,B). Of the 38 kinases studied, the cells transfected with *ACTN4* siRNA showed inactivation of p27 compared to the cells not transfected with *ACTN4* siRNA. No other kinases showed a difference in phosphorylation.

### 3.10 Effects of WAVE2 and ACTN4 on p27 activity

**Figure 10:**
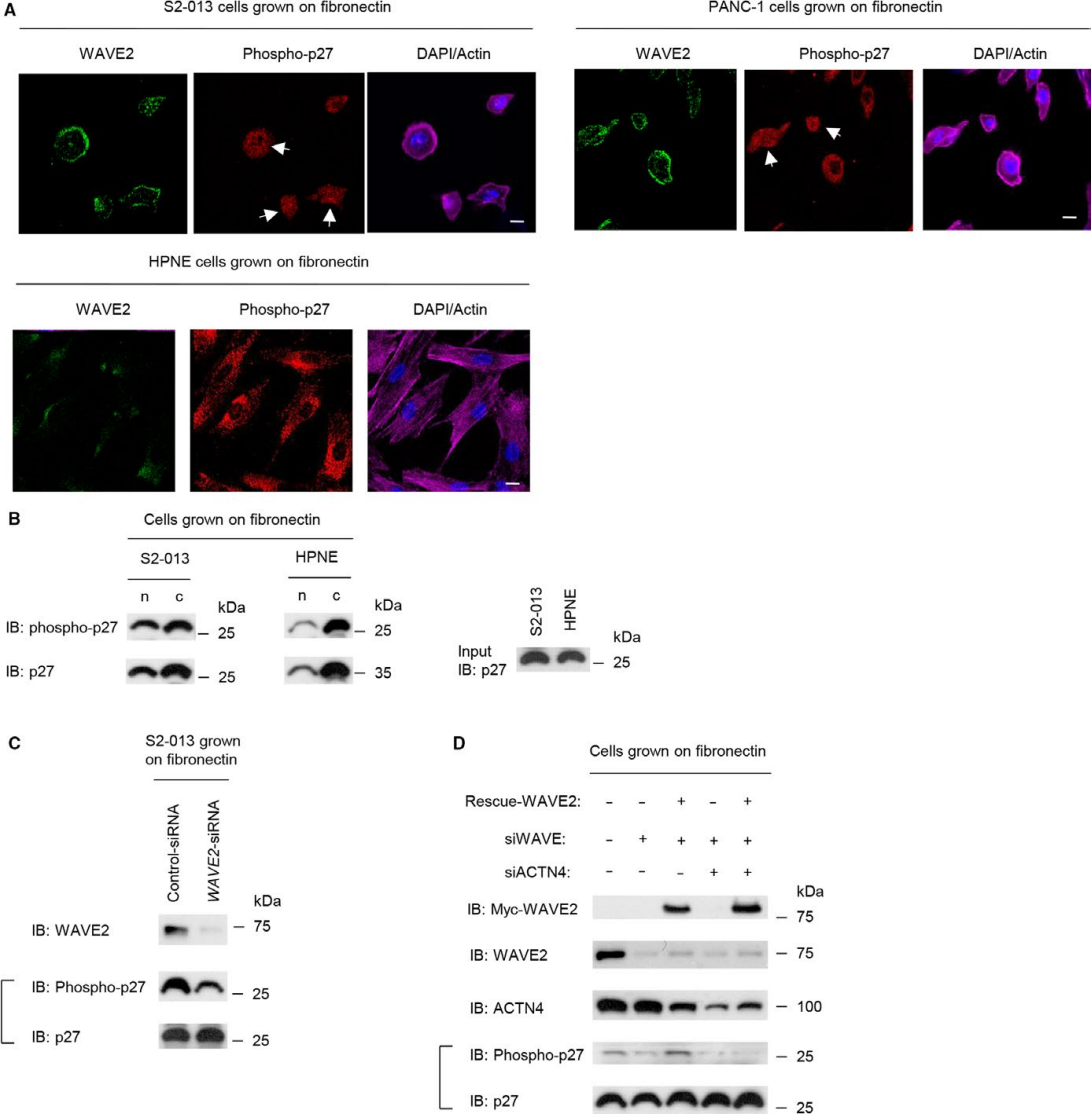
Effects of WAVE2 and ACTN4 on p27 activity. A, Confocal immunofluorescence microscopic images. S2‐013 and HPNE cells were cultured on fibronectin and then labeled with anti‐WAVE2 (green) and anti‐phosphorylated p27 (red) antibodies. Actin filaments were labeled with phalloidin (violet). Arrows, phosphorylated p27 in the nucleus. Blue, DAPI staining. Scale bar, 10 μm. B, S2‐013 and HPNE cells were incubated on fibronectin and fractionated into cytosolic (c) and nuclear (n) fractions. Western blotting of the fractions was performed using anti‐phosphorylated p27 and anti‐p27 antibodies. C, Scr‐transfected S2‐013 cells and siWAVE2‐transfected S2‐013 cells were incubated on fibronectin. Western blotting was performed using anti‐WAVE2, anti‐phosphorylated p27, and anti‐p27 antibodies. D, A myc‐tagged WAVE2 rescue construct was transfected into S2‐013 cells that had been transfected with scrambled control siRNA or *WAVE2* siRNA with or without *ACTN4* siRNA; 48 h later, the cells were incubated on fibronectin. Western blotting was performed using the indicated antibodies

Immunocytochemistry showed that phosphorylated p27 was mainly present in the cytoplasm, and it was also localized in the nucleus of S2‐013 and PANC‐1 cells (Figure 10A). In HPNE cells, phosphorylated p27 was less abundant in the nucleus (Figure 10A). Next, we prepared cellular fractions, and the amount of phosphorylated p27 in these fractions was analyzed by immunoblotting. Phosphorylated p27 was present in the cytosolic and nuclear fractions in S2‐013 cells, although it was obviously reduced in the nuclear fraction of HPNE cells (Figure 10B).

Immunoblotting showed that the suppression of WAVE2 markedly decreased the level of phosphorylated p27 compared to S2‐013 cells transfected with the scrambled control siRNA (Figure 10C). Transfection of a WAVE2 rescue construct into *ACTN4* siRNA‐transfected S2‐013 cells in which WAVE2 had been suppressed did not increase the level of phosphorylated p27 (Figure 10D). In contrast, *WAVE2* siRNA‐transfected S2‐013 cells expressing the rescue WAVE protein showed an increased level of phosphorylated p27 (Figure 10D). These results indicated that WAVE2 and ACTN4 were necessary for regulation of p27 phosphorylation.

### 3.11 Association of p27 with actin polymerization

**Figure 11:**
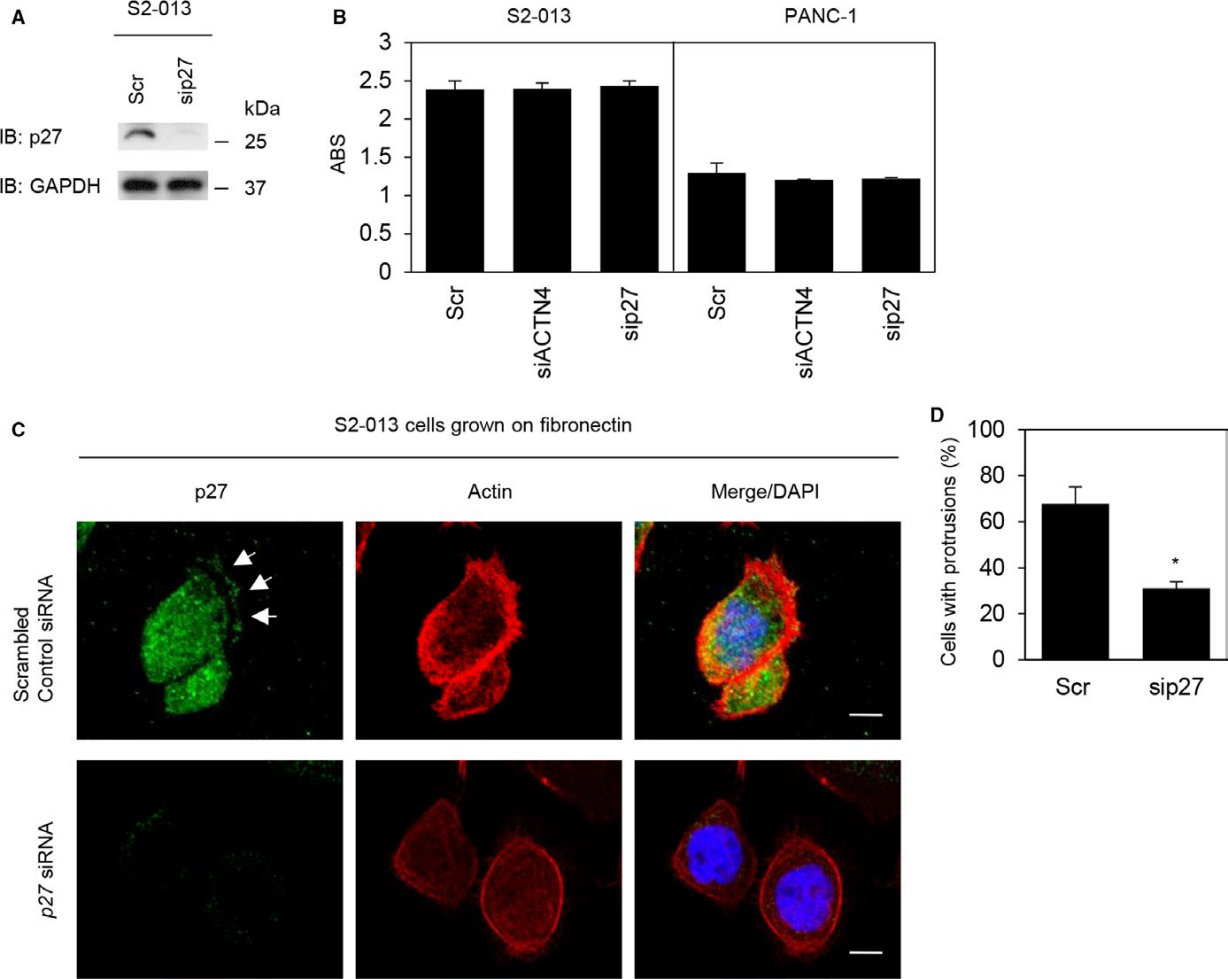
Association of p27 with peripheral rearrangements of the actin cytoskeleton. A, siRNA oligonucleotides targeting *p27* (sip27) or scrambled control siRNAs (Scr) were transiently transfected into S2‐013 cells. Western blotting was performed using anti‐p27 antibody. B, MTT assays of S2‐013 and PANC‐1 cells transiently transfected with scrambled control siRNA, *ACTN4* siRNA, or *p27* siRNA were performed to evaluate cell viability. Data are representative of three independent experiments and are the means ±SD. ABS on *Y*‐axis means absorbance at 490 nm and at 630 nm as reference measured with a microplate reader. C, Confocal immunofluorescence microscopic images. Scr‐transfected S2‐013 cells and sip27‐transfected S2‐013 cells were incubated on fibronectin and subsequently stained with anti‐p27 antibody (green) and phalloidin (red). Arrows, peripheral actin structures in cell protrusions. Blue, DAPI staining. Scale bars, 10 µm. D, Quantification of the data shown in B. The values represent the number of cells with fibronectin‐stimulated cell protrusions in which the levels of peripheral actin structures were increased. All cells in four fields per group were scored. Data were derived from three independent experiments. *Columns*, mean; *bars*, SD. *P < 0.01 compared to the Scr‐transfected control cells (Student's *t* test).

Following transfection with *p27* siRNA or the scrambled control, the level of p27 in S2‐013 cells was assessed by Western blotting (Figure 11A). The p27 level was markedly reduced following *ACTN4* siRNA silencing compared with the scrambled control. p27 is a member of the Cip/Kip family of cyclin‐dependent kinase inhibitors that function to negatively control cell cycle progression.^30^ Overexpression of p27 leads to inhibition of cell proliferation and migration of PDAC cells.^31^ Similar to the results in Figure 4B, MTT assays showed that the number of S2‐013 and PANC‐1 cells transfected with *ACTN4* siRNA or *p27* siRNA was not different compared with scrambled control siRNA‐transfected S2‐013 and PANC‐1 cells (Figure 11B). Suppression of p27 decreased the level of actin polymerization and the formation of cell protrusions compared to control siRNA‐transfected S2‐013 cells (Figure 11C,D). These results indicated that p27 induced the actin‐driven membrane protrusions in PDAC cells, but p27 did not play a role in PDAC cell viability.

### 3.12 Association of WAVE2 with p27 in the promotion of the motility and invasiveness of PDAC cells

**Figure 12:**
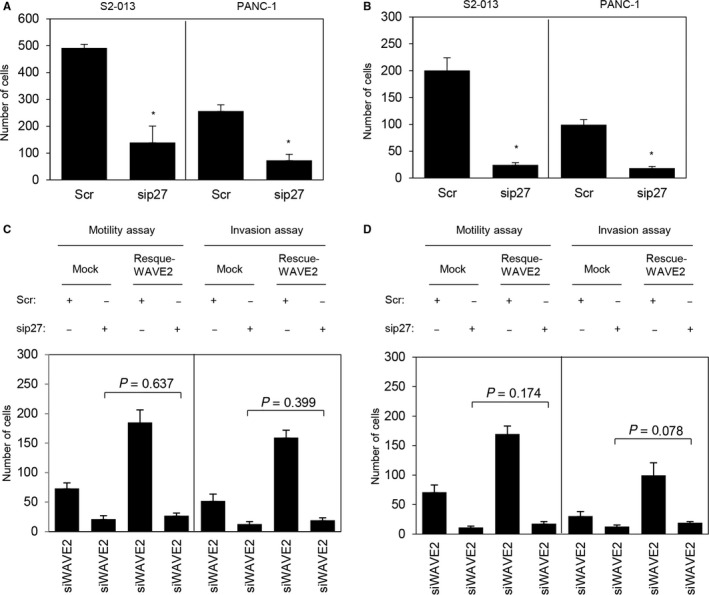
Association of WAVE2 with p27 in the regulation of the motility and invasiveness of PDAC cells. A and B, siRNA oligonucleotides targeting *p27* (sip27) or scrambled control siRNAs (Scr) were transiently transfected into S2‐013 and PANC‐1 cells. Motility (A) and two‐chamber invasion (B) assays were performed. Migrating cells in four fields per group were scored. Data were derived from three independent experiments. *Columns*, mean; *bars*, SD. *P < 0.005 compared to the Scr‐transfected control (Student's *t* test). C and D, A WAVE2 rescue construct was transfected into *WAVE2* siRNA*‐*transfected S2‐013 (C) and *WAVE2* siRNA*‐*transfected PANC‐1 (D) cells that had been transfected with or without *p27* siRNA; 48 h later, motility and two‐chamber invasion assays were performed. Migrating cells in four fields per group were counted. Data were derived from three independent experiments. *Columns*, mean; *bars*, SD.

Suppression of p27 significantly inhibited cell motility and invasion in S2‐013 and PANC‐1 cells (Figure 12A,B). Transfection of a WAVE2 rescue construct into *p27* siRNA‐transfected S2‐013 and PANC‐1 cells in which WAVE2 had been suppressed did not restore cell motility and invasion after silencing of WAVE2 and p27 (Figure 12C,D). These results indicated that WAVE2 and p27 cooperatively promote the motility and invasiveness of PDAC cells.

## 4 DISCUSSION

The present study showed that high WAVE2 expression is closely associated with poor prognosis of patients with PDAC. Coexpression of WAVE2 and Arp2 predicts a poor outcome in patients with invasive breast cancer and is closely associated with aggressive and invasive morphology.^32^ WAVE2 is a candidate prognostic marker in liver cancer and is correlated with a poor prognosis.^33^ In these reports, the intensity of WAVE2 staining in breast cancer or liver cancer specimens was scored without reference to normal cells. The present study is the first report of immunohistochemical analysis of WAVE2 to investigate whether expression of this protein could accurately predict the prognosis of PDAC patients. The intensity of WAVE2 staining in PDAC cells was scored compared to the intensity of the islet of Langerhans as described in previous reports [19, 22). The overall expression score of WAVE2 was not significantly correlated with the clinicopathological variables. Univariate and multivariate Cox regression analyses revealed that high WAVE2 expression was an independent predictor of a worse survival outcome. In vitro motility and invasion assays showed that WAVE2 specifically promoted an invasive phenotype by inducing the formation of cell protrusions in PDAC cells. Our results indicate that WAVE2 may be a determinant of poor prognosis of PDAC patients and is functionally associated with cell motility and invasion.

We recently reported that cytoplasmic RNA granules containing insulin‐like growth factor‐2 mRNA‐binding protein 3 (IGF2BP3)‐bound messenger RNAs (mRNAs) accumulate in the cell protrusions of PDAC cells.^34^, ^35^ Locally translated IGF2BP3‐bound mRNAs in PDAC cell protrusions induce the formation of cell protrusions, thereby promoting invasiveness and metastasis.^34^, ^35^ *WAVE2* mRNA is one of the IGF2BP3‐bound mRNAs in PDAC cells.^34^ Thus, these reports suggest that WAVE2 protein that is concentrated in protrusions may promote cell motility and invasiveness of PDAC cells. Pak1 is required for KIF5B‐mediated transport of a large WAVE2 protein complex toward membrane protrusions in response to hepatocyte growth factor in breast cancer cells.^11^, ^36^ Also, stathmin/Op18 is associated with hepatocyte growth factor‐induced WAVE2 transport to membranes and the formation of cell protrusions.^37^ The WAVE2‐Arp2/3 complex induces the nucleation of actin assembly, thereby leading to the formation of cell protrusions.^5^, ^38^ Thus, WAVE2 is involved in protrusion formation and promotes cell motility and invasion. The present study showed that WAVE2 co‐localized with ACTN4 in the cell protrusions of PDAC cells. ACTN4 is an actin‐binding protein that is abundantly localized in membrane protrusions, such as lamellipodia and filopodia,^39^ but not in cell‐cell junctions.^13^ The expression of ACTN4 in the cytoplasm is associated with worse survival after resection of PDAC and increased lymph node metastases in PDAC.^18^ Knockdown of ACTN4 by siRNAs in PDAC cells reduces motility and invasiveness^17^ and inhibits the formation of microvilli,^13^ indicating that ACTN4 may play integral roles in protrusion formation, motility, and invasion of PDAC cells. In this study, subcellular localization of ACTN4 in PDAC cells with enhanced staining in cell protrusions indicated that WAVE2 functioned by associating with actin filaments via linking with ACTN4 in cell protrusions of PDAC cells. Similar to WAVE2, knockdown of ACTN4 consistently suppressed the formation of cell protrusions driven by actin polymerization and inhibited the motility and invasion of PDAC cells. Our study showed that WAVE2 and ACTN4 cooperatively induced actin‐driven membrane protrusions and promoted the motility and invasiveness of PDAC cells.

Recently, the Rac1/WAVE2/Arp2/3 signaling pathway was reported to promote cell migration and invasion of glioma cells and liver cancer cells.^40^, ^41^ The WAVE2/Annexin A2/DOCK3/β‐catenin signaling pathway promotes the invasiveness and metastasis of liver cancer cells.^42^ These findings indicated that WAVE2‐associated signaling pathways play important roles in cell invasion and metastasis in various types of cancer cells. The present study provides novel evidence that the WAVE2/ACTN4 pathway may be associated with regulation of p27 activity and that this WAVE2/ACTN4/p27 pathway plays important roles in actin polymerization as well as cell motility and invasiveness. Low p27 is correlated with poor prognosis of PDAC.^43^ Although nuclear p27 induces cell cycle arrest and apoptosis, p27 functions as an oncogene by promoting cell migration and metastasis when p27 is mislocalized to the cytoplasm.^44^, ^45^ Phosphorylated p27 immunoreactivity was observed predominantly in the cytoplasm and was present in the nucleus of S2‐013 and PANC‐1 cells, whereas its expression in the nucleus of HPNE cells was lower than in PDAC cells (Figure 10A). Cytoplasmic p27 associates with RhoA and interferes with GDP exchange from RhoA, leading to the formation of actin‐driven membrane protrusions that promote cell migration.^46^ In addition to regulation of RhoA activity, p27 phosphorylation is associated with a signal transducer and activator of transcription 3‐driven metastatic cascade through its association with phosphatidylinositol 3‐kinase activation.^47^ The present study showed that WAVE2 and ACTN4 may be necessary for regulating the phosphorylation of cytoplasmic p27 in PDAC cells and that this WAVE2/ACTN4/p27 pathway plays a role in the formation of actin‐driven membrane protrusions as well as cell motility and invasiveness.

In conclusion, WAVE2 promotes cell motility and invasion and may be a useful marker for poor prognosis of postoperative PDAC patients. Mechanistically, we provided the first evidence that WAVE2 mediates cell motility and invasion by modulating cytoplasmic p27 activity in PDAC cells through linking with ACTN4. In addition, this study indicated that inhibition of (a) WAVE2, (b) the binding of WAVE2 and ACTN4, (c) the binding of ACTN4 and actin filaments, or (d) some combination thereof may be effective for development of new therapeutic strategies, because any such therapy would inhibit WAVE2‐mediated cell motility and invasion of PDAC cells.

## CONFLICT OF INTEREST

The authors have declared that no competing interests exist.
